# Identification of novel *PKD1* and *PKD2* mutations in Korean patients with autosomal dominant polycystic kidney disease

**DOI:** 10.1186/s12881-014-0129-y

**Published:** 2014-12-10

**Authors:** Rihwa Choi, Hayne Cho Park, Kyunghoon Lee, Myoung-Gun Lee, Jong-Won Kim, Chang-Seok Ki, Young-Hwan Hwang, Curie Ahn

**Affiliations:** Department of Laboratory Medicine and Genetics, Samsung Medical Center, Sungkyunkwan University School of Medicine, (135-710) 81 Irwon-Ro Gangnam-gu, Seoul, South Korea; Department of Internal Medicine, Seoul National University College of Medicine, (110-744) 28 Yeongeon-dong, Jongno-gu, Seoul, South Korea; Department of Internal Medicine, Eulji General Hospital, (139-872), 1306 Dunsan 2(i)-dong, Seo-gu, Daejeon, Seoul, South Korea

**Keywords:** Autosomal dominant polycystic kidney disease, Korean, Mutation, *PKD1*, *PKD2*

## Abstract

**Background:**

Autosomal dominant polycystic kidney disease (ADPKD) is the most common inherited kidney disorder. It is caused by mutations in the *PKD1* and *PKD2* genes, and manifests as progressive cyst growth and renal enlargement, resulting in renal failure. Although there have been a few studies on the frequency and spectrum of mutations in *PKD1* and *PKD2* in Korean patients with ADPKD, only exons 36–46, excluding the duplicated region, were analyzed, which makes it difficult to determine accurate mutation frequencies and mutation spectra.

**Methods:**

We performed sequence analysis of 20 consecutive unrelated ADPKD patients using long-range polymerase chain reaction (PCR) to avoid pseudogene amplification, followed by exon-specific PCR and sequencing of the all exons of these two genes. Multiplex ligation-dependent probe amplification was performed in patients in whom pathogenic mutations in *PKD1* or *PKD2* were not identified by LR-PCR and direct sequencing to detect large genomic rearrangements.

**Results:**

All patients met the diagnostic criteria of ADPKD, and pathogenic mutations were found in 18 patients (90.0%), comprising 15 mutations in *PKD1* and three in *PKD2*. Among 10 novel mutations, eight mutations were found in the *PKD1* gene while two mutations were found in the *PKD2* gene. Eight of 14 *PKD1* mutations (57.1%) were located in the duplicated region.

**Conclusions:**

This study expands the spectra of mutations in the *PKD1* and *PKD2* genes and shows that the mutation frequencies of these genes in Korean ADPKD patients are similar to those reported in other ethnicities. Sequence analysis, including analysis of the duplicated region, is essential for molecular diagnosis of ADPKD.

## Background

Autosomal dominant polycystic kidney disease (ADPKD) is the most common inherited kidney disorder, affecting approximately 1 in 400 to 1,000 individuals in Western countries. It is characterized by progressive cyst growth and renal enlargement, resulting in end stage renal disease (ESRD) [1]. ADPKD is caused by mutations in the *PKD1* (16p13.3) and *PKD2* (4q21) genes, which produce the proteins polycystin-1 (PC-1) and PC-2, respectively. These proteins have critical roles in maintaining normal renal tubular structure during kidney development. These proteins have also been found in primary cilia, where they function as mechanosensors between cells, or at cell-matrix attachments, where they mediate cell adhesion [2]. In clinically defined populations, mutations in *PKD1* and *PKD2* account for ~85% and ~15% of ADPKD cases, respectively [1,3]. Patients with *PKD1* mutations exhibit a more progressive renal phenotype than those with *PKD2* mutations (average age at onset of ESRD, 53.4 years vs. 72.7 years, respectively) [4].

ADPKD is typically diagnosed by renal imaging with age-related cyst number criteria; however, imaging cannot exclude disease in at-risk individuals under 40 years of age [5,6]. Mutation-based diagnostics are increasingly being used in individuals who are at risk of ADPKD, and have proven especially helpful in cases where imaging studies are equivocal and a definitive diagnosis is required, such as when evaluating potential living related kidney donors [7]. In addition, *PKD1* and *PKD2* mutation analyses can potentially provide prognostic as well as diagnostic information, such as pre-implantation genetic diagnostics for early-onset ADPKD with the advent of effective therapies for ADPKD [7,8].

Mutation analysis of ADPKD has been hampered by the large sizes and multi-exon structures of *PKD1* and *PKD2*, genomic duplication of *PKD1*, marked allelic heterogeneity, and common missense variants with hypomorphic alleles [7]. Two-thirds of the *PKD1* gene (exons 1–33) is duplicated six times on chromosome 16 (pseudogenes *PKD1P1-P6*), which share 97.7% sequence identity with *PKD1* but carry large deletions compared with *PKD1* [9]. To analyze exons 1–33 of *PKD1*, long-range PCR (LR-PCR) needs to be performed before sequencing procedures [10]. Although several indirect methods have been developed, such as denaturing gradient gel electrophoresis (DGGE), single-strand conformation polymorphism (SSCP) analysis, protein-truncation tests, denaturing high performance liquid chromatography (DHPLC), transgenomics SURVEYOR nuclease and WAVE nucleic acid high sensitivity fragment analysis, and high resolution melt analysis, direct sequencing is undoubtedly the gold standard [3]. Quantitative PCR, multiplex ligation-dependent probe amplification (MLPA), and array-comparative genomic hybridization (array-CGH) can also aid in the detection of large genomic rearrangements [3,11].

Studies using different molecular diagnostic methods have reported different mutation detection rates among different populations [1,3,12-17]. Although there are a few studies on the frequency and spectrum of mutations in the *PKD1* and *PKD2* genes in Korean patients with ADPKD, only exons 36–46 of *PKD1* were analyzed, making it difficult to determine accurate mutation frequencies and spectra [18-20]. In this study, we performed LR-PCR followed by nested PCR and direct sequencing as well as MLPA of *PKD1* and *PKD2* in 20 unrelated Korean ADPKD patients, covering all exons and flanking intronic regions for the first time.

## Methods

We analyzed 20 unrelated patients with ADPKD from April 2010 to April 2014. The diagnosis of ADPKD was made according to previously described renal ultrasound criteria, including a family history of ADPKD [5,6]. Blood samples were obtained after receiving informed consent. This study was approved by the Institutional Review Board of Samsung Medical Center.

Genomic DNA was extracted from peripheral blood samples using a commercially available extraction kit (Promega, Madison, WI, USA). For the duplicated region of *PKD1*, LR-PCR was performed using *PKD1*-specific primers as described previously [21]. Direct sequencing of exonic and flanking introgenic regions of all *PKD1* and *PKD2* exons was performed using previously-described PCR primers and conditions [21]. PCR products were purified and sequenced on an ABI3730*xl* Genetic Analyzer (Applied Biosystems, Foster City, CA, USA).

MLPA was performed in patients in whom pathogenic mutations were not identified in *PKD1* or *PKD2* by LR-PCR and direct sequencing to detect large genomic rearrangements. Deletion/duplication analysis of *PKD1/PKD2* exons was performed using the MLPA kit (SALSA MLPA KIT P351-B2/P352-C1 PKD1-PKD2; MRC-Holland, Amsterdam, The Netherlands) according to the manufacturer’s instructions. This kit contains *PKD1* probes for 37 of the 46 exons and covers all 15 *PKD2* exons.

The 1000 Genomes Project data (http://browser.1000genomes.org), the National Heart, Lung, and Blood Institute (NHLBI) Exome Sequencing Project (ESP) database (http://evs.gs.washington.edu/EVS), the NCBI database of Single Nucleotide Polymorphisms (dbSNPs), and the ADPKD Mutation Database (http://pkdb.mayo.edu, PKDB) were checked for previously reported sequence variants.

The pathogenicity of missense variants was evaluated by *in silico* analyses using Sorting Intolerant from Tolerant (SIFT) (http://sift.jcvi.org/), Polymorphism Phenotyping v.2 (PolyPhen-2) (http://genetics.bwh.harvard.edu/pph2/), and the Grantham matrix scoring system Align Grantham Variation and Grantham Deviation (Align-GVGD) (http://agvgd.iarc.fr/) prediction programs, as described previously [1]. Human Splice Finder software (http://umd.be/HSF/) was used to predict splicing signals [22].

Variations identified in the current study were categorized into five classes: definite pathogenic mutation, likely pathogenic mutation, variant of unknown significance (VUS), likely benign variant, and benign [23]. The first class refers to nonsense mutations, frameshift deletions, insertions or indels, typical splicing mutations, or in-frame changes of ≥ 5 amino acids detected by direct sequencing, as well as large genomic rearrangements detected by MLPA. In-frame changes of < 5 amino acids, as well as missense and atypical splicing variants previously reported in patient(s) with sufficient evidence to classify them as pathogenic were also classified as definite pathogenic mutations. The second class refers to novel missense variants with the potential of pathogenicity as evaluated by *in silico* analyses with agreement among various methods, which included ‘Not tolerated’ on SIFT, ‘Probably damaging’ or ‘Possibly damaging’ on PolyPhen-2, and a score of C55 to C66 on Align-GVGD. Variations previously reported as ‘likely pathogenic’, but without sufficient evidence, were also classified as the second class. The third class refers to novel variants (synonymous or missense variants) predicted not to affect protein function by any of the *in silico* analyses, and intronic variations predicted not to alter splicing that were absent from the 1000 Genome Project data, ESP database of more than 13,000 control alleles, and an in-house collection of 150 exomes, or previously reported variations termed ‘indeterminate’ on PKDB. The fourth class refers to novel synonymous variations not reported previously that were found in control studies, suggesting that they are rare benign polymorphisms. The last class refers to benign SNPs. The recommendations of the Human Genome Variation Society for mutation nomenclature were followed. Nucleotide changes were numbered according to the coding sequences of *PKD1* (NM_001009944.2) and *PKD2* (NM_000297.3).

## Results

During the 4-year study period, we performed mutational analysis using LR-PCR, followed by nested PCR and direct sequencing of *PKD1* and *PKD2* in 20 unrelated Korean patients diagnosed with ADPKD by ultrasound. Four of 20 PKD patients with an unknown or no family history of polycystic kidney disease had enlarged kidneys with more than 10 cysts in each kidney. There were 15 male and five female patients. The mean (± S.D.) age was 42.6 (± 8.4) years.

We identified 20 different variants in *PKD1* and *PKD2* among 20 ADPKD patients, including 17 pathogenic sequence variants, two VUS identified by sequence analysis, and one large deletion identified by MLPA analysis. Among these variants, 18 were classified as pathogenic mutations (7 frameshift, 6 nonsense, 3 missense, 1 splicing, and 1 large deletion). For six of seven patients who were not identified as having definite pathogenic mutations in sequence-based mutation analysis, MLPA analyses were performed. In one patient who was identified as having a likely pathogenic mutation of c.11541C > G (p.Ser3847Arg) in *PKD1*, MLPA analysis could not be performed because additional samples were not available. Each ADPKD patient had a unique mutation in *PKD1* or *PKD2*. We found disease-causing mutations in patients with an overall mutation detection rate of 90.0% (18/20); 94.4% (17/18) patients were identified by LR-PCR and direct sequencing. The remaining one patient (5.6%) was identified as having a *PKD1* mutation with large genomic rearrangements (deletion of one copy of the whole *PKD1* gene) by MLPA.

Among the 18 patients shown to have pathogenic mutations, 15 (83.3%) patients had mutations in *PKD1* (6 frameshift, 4 nonsense, 3 missense, 1 splicing, and 1 large deletion) and three (16.7%) patients had two nonsense and one frameshift *PKD2* mutations (Tables 1 and 2). Thirteen of the 18 (72.2%) pathogenic mutations were nonsense or frameshift mutations: 66.7% (10/15) in *PKD1* and 100% (3/3) in *PKD2*. All three missense variants found in *PKD1* were classified as likely pathogenic mutations and were predicted to affect protein function by all three *in-silico* analyses. Ten of 18 (55.6%) mutations were not previously reported, including eight mutations in the *PKD1* gene and two mutations in the *PKD2* gene. Among 14 *PKD1* mutations identified by LR-PCR and direct sequencing, eight (57.1%) mutations were located in the duplicated region, outside the range of exons 36–46, and were not covered in previous reported studies in Korea.

**Table 1:** **Definitive pathogenic mutations in** ***PKD1*** **and** ***PKD2*** **found in this study**

**Gene**	**Exon/Intron**	**cDNA change**	**Amino acid change**	**Known/Novel**	**Type**	**Family history**	**Reference**
*PKD1*	12	c.2865delC	p.Val956Trpfs*20	Known	Frameshift	+	PKDB [3]
*PKD1*	12	c.2932C > T	p.Gln978*	Novel	Nonsense	+	
*PKD1*	15	c.4379_4380delTG	p.Val1460Glyfs*62	Novel	Frameshift	+	
*PKD1*	19	c.7579_7580delGT	p.Val2527Leufs*67	Novel	Frameshift	+	
*PKD1*	22	c.8019dupG	p.Pro2674Alafs*148	Novel	Frameshift	+	
*PKD1*	26	c.9340C > T	p.Gln3114*	Novel	Nonsense	+	
*PKD1*	42	c.11545delG	p.Ala3849Leu*96	Novel	Frameshift	+	
*PKD1*	42	c.11614G > T	p.Glu3872*	Known	Nonsense	+	PKDB [21]
*PKD1*	43	c.11766G > A	p.Trp3922*	Known	Nonsense	+	PKDB [1]
*PKD1*	45	c.12155_12156delTG	p.Val4052Glyfs*104	Known	Frameshift	+	PKDB (unpublished)^a^
*PKD2*	2	c.667G > T	p.Glu223*	Novel	Nonsense	+	
*PKD2*	8	c.1888_1891delinsGT	p.Gln630Valfs*20	Novel	Frameshift	+	
*PKD2*	13	c.2407C > T	p.Arg803*	Known	Nonsense	+	PKDB [3]

^a^This mutation has been reported in one family (ATH0279) from unpublished data and is registered in PKDB by Athena Diagnostics.

**Table 2:** **Likely pathogenic mutations found in this study and prediction of their pathogenicity**

**Gene**	**Exon/Intron**	**cDNA change**	**AA change**	**Known/Novel**	**Type**	**SIFT**	**PolyPhen-2**	**GVGD**	**Family history**
						**VS**	**VS**	**VS**	
*PKD1*	26	c.9380G > C^a^	p.Gly3127Ala	Novel	Missense	0.00	0.994	C55	+
*PKD1*	39	c.11248C > G^a^	p.Arg3750Gly	Known^b^	Missense	0.00	0.997	C65	-
*PKD1*	42	c.11541C > G^a^	p.Ser3847Arg	Novel	Missense	0.01	0.805	C65	+
*PKD1*	IVS5	c.1202-9G > A	p.Ala401fs	Known^c^	Splice				-

Abbreviations: AA amino acid, VS variant score, NA not applicable.

^a^The three novel missense variations found in this study were predicted to affect protein function by all three *in-silico* analyses.

^b^This mutation has been reported in two affected patients among four tested family members in one Czech family [27].

^c^This mutation has been reported in one family (ATH0012) from unpublished data by Athena Diagnostics and is registered in PKDB and classified as ‘likely pathogenic’ with the amino acid change of p.Ala401fs. Kurashige et al. also reported that this mutation created a new acceptor site seven nucleotides upstream of the original acceptor site, which was confirmed by a minigene splicing assay [17].

Two novel VUSs that were identified in patients without known PKD mutations are summarized in Table 3. The novel intronic variation of c.595 + 15C > T, which was found in *PKD2,* was predicted to have no effect on normal splicing but was not identified in healthy controls from the 1000 Genome Project data, the ESP database of more than 13,000 control alleles, or an in-house collection of 150 exomes. Another novel synonymous variant of c.11604G > C (p.Thr3868=) was found in *PKD1* exon 42. The question of whether this variant is a rare SNP or a disease-associated pathogenic variant could not be easily addressed; although its effect on mRNA splicing was not examined *in vitro*, HSF predicted it would affect an enhancer motif.

**Table 3:** **Novel variants with unknown clinical significance found in this study**

**Gene**	**Exon/Intron**	**cDNA change**	**AA change**	**Known/Novel**	**HSF prediction**	**Family history**
*PKD1*	42	c.11604G > C^a^	p.Thr3868=	Novel	Potentially affects enhancer motif	+
*PKD2*	IVS1	c.595 + 15C > T	?	Novel	No effect on normal splicing	-

Neither variations were observed in healthy controls in this study; HSF, Human Splice Finder software (http://umd.be/HSF/) used to predict splicing signals.

^a^This variant was observed in one patient with a family history of polycystic kidney disease (mother). Unfortunately, molecular diagnostic test results for his affected family member were not available.

## Discussion

To the best of our knowledge, this is the first study to identify *PKD1* and *PKD2* mutations based on analysis of all coding exons and flanking regions in Korean ADPKD patients using LR-PCR followed by direct sequencing and MLPA.

The mutation detection rate (90.0%) in our study (83.3% in *PKD1* and 16.7% in *PKD2*) was comparable to previous studies using LR-PCR followed by nested PCR and direct sequencing in clinically well-defined large cohorts in Western countries [1,3]. Although the proportion of *PKD2* mutations has been reported to be ~23% in Asian populations [16,17], *PKD2* mutations accounted for 16.7% of the total mutations in our study subjects, comparable to data from Western countries (15.0%-16.2%) [1,3]. In contrast, a recent population-based study (Olmsted County population study) [24] and studies in an outpatient clinic setting [25,26] reported a higher *PKD2* mutation detection rate than earlier studies, which recruited large families with a history of ERSD. Future studies of a larger cohort of patients are needed to further understand the influence of ethnicity on genetic and/or phenotypic characteristics of ADPKD patients.

Among 14 pathogenic *PKD1* mutations (excluding one large gene deletion), eight (57.1%) were located outside the range of exons 36–46. The low mutation detection rate previously reported in Korean ADPKD patients could be explained by the fact that different mutational analyses techniques were used. In studies on other ethnic populations with different molecular diagnostic methods, mutations within *PKD1* or *PKD2* were detected in 63.0-89.9% of patients from Western populations [1,3,12-14,27], and 52.3-83.9% of patients from Asian populations outside of Korea [15-17] (Table 4).

**Table 4:** **Different mutation detection rates among different study groups with different diagnostic methods**

**Ethnicity**	**No. of patients** ^**a**^	**Mutation detection rate**	**Method**	**Reference**
**Caucasian**				
American	202	89.1%	DHPLC, LR-PCR, DS	[1]
American	183	63.0%	LR-PCR, NGS, targeted re-sequencing	[12]
German	93	64.5%	LR-PCR, DS	[13]
German	277	64.6%	DHPLC, LR-PCR, DS	[14]
Czech	56	71.0%	Linkage analysis, LR-PCR, nested PCR with HRM, DS	[27]
French	700	89.9%	LR-PCR, DS, QFM PCR or Array-CGH	[3]
**Asian**				
Chinese Hans	65	52.3%	DHPLC, LR-PCR, DS	[15]
Taiwanese	46	65.0%	LR-PCR, DS, and Real time Q-PCR or MLPA	[16]
Japanese	161	83.9%	LR-PCR, DS, and Q-PCR or MLPA	[17]
Korean	20	90.0%	LR-PCR, DS, and MLPA	This study

Abbreviations: DHPLC, denaturing high performance liquid chromatography; LR-PCR, long range-PCR; DS, direct sequencing; NGS, next-generation DNA sequencing; HRM, high resolution melting; QFM PCR, quantitative fluorescent multiplex PCR; Array-CGH, array-comparative genomic hybridization; Q-PCR, quantitative PCR; MLPA, multiplex ligation-dependent probe amplification.

^a^Number of unrelated patients in the study group.

In previous studies on Korean ADPKD patients, methods other than LR-PCR and direct sequencing were used to detect mutations in *PKD1* and *PKD2*, such as PCR and SSCP analysis for *PKD1* exons 36–46 excluding the duplicated region. This approach had a mutation detection rate ranging from 6.6% (6/91 unrelated individuals) [18] to 13.7% (7/51 families) [20]. Other methods previously used include two-dimensional gene scanning followed by DGGE and direct sequencing of *PKD2*; a mutation detection rate of 13.0% (6/46 patients) was reported using this approach [28]. Linkage analysis using PCR and polyacrylamide gel electrophoresis for *PKD1* and *PKD2* resulted in a mutation detection rate of 81.3% (39/48 families) [19]. However, linkage analysis is not suitable for small families or families with a single affected individual, and becomes complicated if a *de novo* mutation has recently occurred in the family.

In this study, each patient had a unique pathogenic mutation; no mutations were repeatedly found in more than one individual, suggesting that a recurrent or founder mutation is not likely in this study group. This is consistent with the findings that no single mutation accounted for >2% of all unrelated ADPKD patients in previous studies [3,16]. These findings support the suggestion that Korean ADPKD patients who seek a genetic diagnosis need to undergo complete mutation analyses of *PKD1* and *PKD2* unless causative mutations have been identified in their family members.

MLPA should be reserved for those patients in whom a mutation has not been identified by LR-PCR and direct sequencing. Large genomic rearrangements have rarely been reported, and account for <2% of the total pathogenic mutations in the *PKD1* gene according to the ADPKD mutation database (PKDB). In our study, we identified one patient with a heterozygous deletion of entire *PKD1* gene. This large deletion was not characterized at the nucleotide level due to the sequence complexity of the *PKD1* locus.

Pathogenic mutations were not found in two of 20 patients (10.0%) in this study, which is comparable to the results reported for a Japanese cohort (16.1%) [17] and Western countries (10.1%-10.9%) [1,3]. There are several possible reasons for this finding. First, it is possible that mutations occurred within deep intronic regions, as well as in promoters and other distantly located regulatory regions not covered by the current exon-based sequencing method. Second, some of the variants that were classified as other than pathogenic mutations may in fact be hypomorphic variants with incomplete penetrance. Such variants may cause mild cystic disease, but two such variants in trans could cause typical to severe disease [7]. Otherwise, further genetic heterogeneity such as mosaicism or mutations in other genes such as *HNF1β, PRKCSH, SEC63*, or *PKHD1* may contribute to the cystic phenotype [7]. One of the two patients in whom no pathogenic mutations in *PKD1* or *PKD2* were found, except for the synonymous variant of c.11604G > C (p.Thr3868=) in *PKD1*, had a history of multiple liver cysts that developed in early childhood, but relatively small renal cysts in adulthood, which suggests that genes other than *PKD1* and *PKD2* could be involved in pathogenicity in this individual. However, this patient had a positive family history (mother with PKD), indicating that this variant could be a novel pathogenic mutation that could potentially affect splicing. Unfortunately, mutational analysis of the patient’s affected mother was not performed. The other patient without identified pathogenic mutations aside from one intronic VUS (c.595 + 15C > T) in *PKD2* had no family history.

As potential therapies and clinical trials with different mechanism-based drug treatments for ADPKD are being developed, more efficient, accurate, and low-cost genetic testing is needed and expected in the next decade with the advance of DNA sequencing techniques [29]. Considering the mutation spectrum in Korean patients with ADPKD, LR-PCR followed by nested PCR and direct sequence analysis of the duplicated region should be performed for an accurate molecular diagnosis and to determine the appropriate course of treatment for the disease.

The limitations of this study include the relatively small sample size, which limited our power to draw meaningful statistical conclusions regarding mutation prevalence and mutation detection rate. In addition, there was limited clinical data regarding genotype-phenotype correlations, and lack of experimental functional validation of novel variations. Future studies are required to investigate the significance of the VUS identified in the study.

## Conclusions

*PKD1* and *PKD2* mutations were detected in 90.0% of Korean ADPKD patients; mutations in *PKD1* and *PKD2* accounted for 83.3% and 16.7% of all mutations, respectively. Of the 14 mutations found in *PKD1* (except for one large gene deletion), eight (57.1%) mutations were located outside the range of exons 36–46. Considering the mutation spectrum in Korean patients with ADPKD, LR-PCR followed by direct sequence analysis of the duplicated region should be performed for accurate molecular diagnosis of this disease.

## Abbreviations

ADPKD: Autosomal dominant polycystic kidney disease
Align-GVGD: Align Grantham Variation and Grantham Deviation, http://agvgd.iarc.fr/
CGH: Comparative genomic hybridization
DGGE: Denaturing gradient gel electrophoresis
DHPLC: Denaturing high performance liquid chromatography
ESP: Exome Sequencing Project
ESRD: End-stage renal disease
HSF: Human Splice Finder software, http://umd.be/HSF/
LR-PCR: Long range polymerase chain reaction
MLPA: Multiplex ligation-dependent probe amplification
NHLBI: National Heart, Lung, and Blood Institute
PC: Polycystin
PCR: Polymerase chain reaction
PKD: Polycystic kidney disease
PKDB: ADPKD Mutation Database, http://pkdb.mayo.edu
PolyPhen-2: Polymorphism Phenotyping v.2, http://genetics.bwh.harvard.edu/pph2/
SIFT: Sorting intolerant from tolerant, http://sift.jcvi.org/
SNP: Single nucleotide polymorphism
SSCP: Single-strand conformation polymorphism
VUS: Variant of unknown significance
